# Postoperative Lung Function after Robotic- versus Video-Assisted Thoracoscopic Lobectomy for Lung Cancer: A Propensity Score-Matched Study

**DOI:** 10.5761/atcs.oa.25-00123

**Published:** 2025-12-16

**Authors:** Hisashi Oishi, Ken Onodera, Hirotsugu Notsuda, Tatsuaki Watanabe, Yui Watanabe, Takaya Suzuki, Hiromichi Niikawa, Takeo Togo, Sakiko Kumata, Yoshinori Okada

**Affiliations:** Department of Thoracic Surgery, Institute of Development, Aging and Cancer, Tohoku University, Sendai, Miyagi, Japan

**Keywords:** robotic-assisted thoracoscopic surgery, video-assisted thoracoscopic surgery, lung cancer, postoperative pulmonary function

## Abstract

**Purpose:**

Robotic-assisted thoracoscopic surgery (RATS) has emerged as an alternative to video-assisted thoracoscopic surgery (VATS) for lobectomy in early-stage non-small cell lung cancer (NSCLC). While perioperative and oncologic outcomes have been studied, limited data exist on long-term postoperative pulmonary function. This study compared pulmonary function one year after RATS versus VATS lobectomy.

**Methods:**

We retrospectively analyzed 298 patients who underwent lobectomy for early-stage NSCLC between September 2020 and August 2023. After applying exclusion criteria, 186 patients remained: 128 in the VATS group and 58 in the RATS group. Propensity score matching (1:1) yielded 55 matched pairs. Pulmonary function parameters—%predicted forced vital capacity (%FVC), %predicted forced expiratory volume in one second (%FEV1), %predicted peak expiratory flow (%PEF), and %predicted diffusing capacity for carbon monoxide (%DLco)—were evaluated one year postoperatively.

**Results:**

No significant differences were observed between groups in any pulmonary function parameters at one year, both before and after matching. Lung function was similarly preserved.

**Conclusions:**

Despite the use of more ports, RATS lobectomy did not result in inferior pulmonary function compared to VATS. Given its higher cost, VATS may remain the more cost-effective standard, although RATS offers a promising platform for future innovation.

## Introduction

Video-assisted thoracoscopic surgery (VATS) is an established standard treatment for early-stage lung cancer in many regions worldwide. Robotic-assisted thoracoscopic surgery (RATS) has also gained broader adoption in recent years, with a growing number of institutions implementing the technique. Numerous studies have compared the outcomes of VATS and RATS in lung resection for early-stage lung cancer. Although RATS lung resection has been associated with higher hospital-related costs than VATS, perioperative and postoperative outcomes have been reported to be comparable for both procedures.^1)^ Louie et al. demonstrated that VATS and RATS lobectomy yielded similar results in terms of postoperative complications, hospital stay duration, 30-day mortality, and nodal upstaging.^2)^ More recently, Alwatari et al. reported that RATS was associated with increased total hospital costs and longer hospital stays, although thoracic complication rates tended to be lower following RATS lobectomy.^3)^ Regarding lymph node retrieval, Mungo et al. noted potential technique-specific advantages with RATS.^4)^ In our findings, RATS reduced mediastinal nodal dissection time during right upper lobectomy with mediastinal nodal dissection.^5)^ Fabbri et al. focused on long-term oncologic outcomes and found that RATS lobectomy resulted in significantly higher disease-free survival and significantly lower recurrence rates compared with VATS.^6)^

In this study, we focused on postoperative lung function. Only a limited number of reports have addressed lung function after RATS lobectomy.^7,8)^ The aim of this study was to compare lung function one year after RATS lobectomy for early-stage lung cancer with that after VATS lobectomy.

## Materials and Methods

### Patients, data collection, and study groups

**Fig. 1:**
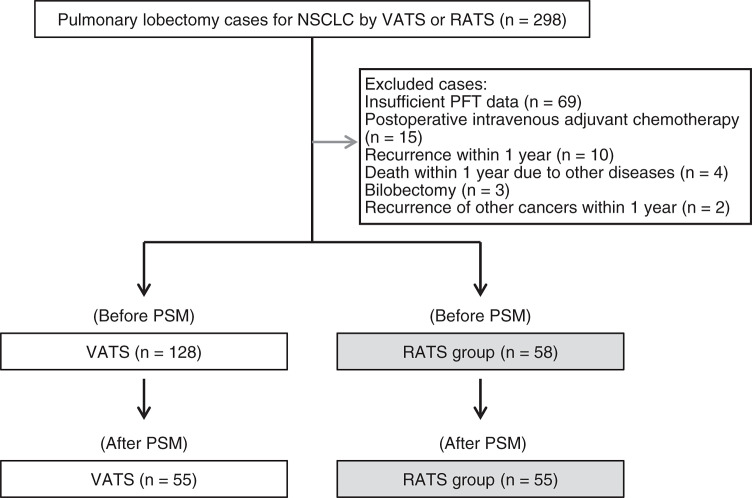
Inclusion and exclusion criteria for study participants. Flowchart illustrating the inclusion and exclusion process for the study cohort. A total of 298 patients who underwent lobectomy for NSCLC were initially screened. After applying exclusion criteria, 186 patients were included in the analysis: 128 in the VATS group and 58 in the RATS group. Following PSM, 55 matched pairs were analyzed. NSCLC: non-small cell lung cancer; VATS: video-assisted thoracoscopic surgery; RATS: robotic-assisted thoracoscopic surgery; PFT: pulmonary function test; PSM: propensity score matching

A retrospective analysis was conducted of 298 patients who underwent pulmonary lobectomy for non-small-cell lung cancer (NSCLC) using either a VATS or RATS approach at the institution between September 2020 and August 2023. A total of 103 patients were excluded for the following reasons: insufficient pulmonary function test (PFT) data (n = 69), administration of postoperative intravenous adjuvant chemotherapy (n = 15), recurrence within one year (n = 10), death from non-cancer-related causes within one year (n = 4), bilobectomy (n = 3), and recurrence of other cancers within one year (n = 2). The final analysis included 186 patients, categorized into two groups: the VATS group, comprising patients who underwent pulmonary lobectomy using the VATS approach, and the RATS group, comprising those treated with the RATS approach (**Fig. 1**). Patients underwent RATS lobectomy when the robotic surgical system and trained personnel were available; otherwise, VATS was performed. Thus, the allocation was primarily based on system availability rather than patient characteristics, approximating a quasi-randomized design. All RATS procedures were performed by surgeons certified in robotic thoracic surgery. The same group of surgeons also performed the VATS procedures, ensuring that surgical experience was comparable between groups. Data on preoperative demographics, surgical and pathological characteristics, and PFT results were collected. PFTs were obtained within two months prior to surgery and at one year postoperatively. Postoperative complications were classified according to the Common Terminology Criteria for Adverse Events (CTCAE), version 5.0,^9)^ or the Clavien–Dindo classification system.^10,11)^ Complications of grade 2 or higher on the CTCAE or grade II or higher on the Clavien–Dindo scale were defined as significant complications.

### Procedures of VATS and RATS

For both approaches, patients were positioned laterally, and rib spreading was not performed.

VATS pulmonary lobectomy was performed using a 4–5 cm utility incision at the fourth or fifth intercostal space (ICS) along the anterior axillary line, a 12-mm camera port at the seventh ICS along the midaxillary line, and a 20-mm incision at the seventh ICS near the scapular tip. A 10-mm, 30-degree camera was used for visualization. Specimens were retrieved through the utility incision. At the end of the procedure, a chest tube was inserted through the port incision at the seventh ICS in the midaxillary line and directed toward the apex of the thoracic cavity.

RATS pulmonary lobectomy was performed using a da Vinci Xi Surgical System (Intuitive Surgical, Sunnyvale, CA, USA). Five port sites were used: a utility incision at the fourth or fifth ICS in the anterior axillary line; an 8-mm camera port at the eighth ICS in the midaxillary line; two 12-mm ports at the seventh ICS (for upper or middle lobectomy) or eighth ICS (for lower lobectomy) in the anterior axillary line and the eighth ICS in the posterior axillary line; and an 8-mm port at the eighth ICS near the scapular tip. An 8-mm, 30-degree camera was used for visualization. Specimens were extracted through the utility incision. The instrumentation included fenestrated bipolar forceps, Maryland bipolar forceps, Cadiere forceps, a vessel sealer, and SureForm staplers. At the conclusion of the procedure, a chest tube was placed through a port incision and directed toward the apex of the thoracic cavity.

### Statistical analysis

Data are presented as mean ± standard error with ranges, or as counts and percentages (n, %). For parametric data, Student’s t-test or Welch’s t-test was used; for nonparametric data, the Mann–Whitney *U* test was applied. Categorical variables were analyzed using Fisher’s exact test or the chi-squared test. To minimize potential selection bias, propensity score matching (PSM) was performed to balance baseline confounding variables between the two groups. A 1:1 matching ratio was achieved using propensity scores and the nearest-neighbor method. Matching variables included sex, age, body mass index (BMI), smoking history, type of lobectomy, pathological type, and pathological stage. Statistical analyses were performed using Prism 5 (GraphPad Software Inc., La Jolla, CA, USA) and JMP Pro 17 (SAS Institute Inc., Cary, NC, USA). A *p*-value <0.05 was considered statistically significant.

## Results

### Patient and clinicopathological characteristics

Of the 186 patients included in the analysis, 128 were assigned to the VATS group and 58 to the RATS group (**Fig. 1**). Before PSM, no significant differences were observed between the groups in age, sex, body weight, BMI, smoking history, performance status, medical history, operative time, blood loss, type of lobectomy, postoperative complications, histological type, or pathological stage (**Table 1**). After PSM, 55 patients were successfully matched in each group (**Fig. 1**). None of the examined parameters differed significantly between the matched groups (**Table 2**). Importantly, postoperative complication rates remained comparable between the VATS and RATS groups both before and after PSM (**Tables 1** and **2**).

**Table 1:** Clinicopathological characteristics before propensity score matching

Characteristic	VATS group (n = 128)	RATS group (n = 58)	*p*-Value
Age (years)	69.1 ± 0.9 (17–87)	69.1 ± 1.2 (47–87)	0.98
Sex, male/female (%)	74 (57.8%)/54 (42.2%)	25 (43.1%)/33 (56.9%)	0.06
Height (cm)	161.2 ± 0.8 (138–184)	158.5 ± 1.2 (142–177)	0.06
Body weight (kg)	61.8 ± 1.0 (39.6–93.5)	59.7 ± 1.5 (36–92.2)	0.25
BMI (kg/m^2^)	23.7 ± 0.3 (17.7–38.2)	23.6 ± 0.5 (14.9–33.0)	0.95
Smoking history, yes/no (%)	78 (60.9%)/50 (39.1%)	29 (50.0%)/29 (50.0%)	0.15
Performance status, 0/1 (%)	120 (93.8%)/8 (6.3%)	57 (98.3%)/1 (1.7%)	0.28
Past medical history			
Hypertension	44 (34.4%)	21 (36.2%)	0.28
Malignant disease	17 (13.3%)	9 (15.5%)	0.64
Arrhythmia	6 (4.7%)	3 (5.2%)	1.00
Ischemic heart disease	4 (3.1%)	2 (3.4%)	1.00
Diabetes mellitus	4 (3.1%)	2 (3.4%)	0.65
Interstitial lung disease	4 (3.1%)	1 (1.7%)	1.00
Cerebrovascular disease	3 (2.3%)	2 (3.4%)	1.00
Operative time (min)	162 ± 4.2 (85–306)	170 ± 5.6 (84–280)	0.33
Blood loss (mL)	35 ± 6.1 (1–565)	39 ± 11.5 (1–522)	0.84
Lobectomy procedure types			0.40
RUL	44 (34.4%)	24 (41.4%)	
RML	10 (7.8%)	1 (1.7%)	
RLL	25 (19.5%)	14 (24.1%)	
LUL	31 (24.2%)	12 (20.7%)	
LLL	18 (14.1%)	7 (12.1%)	
Postoperative complications			
Pulmonary complications	14 (10.9%)	8 (13.8%)	0.63
Cardiovascular complications	5 (3.9%)	3 (5.2%)	0.71
Histologic type			0.69
Adenocarcinoma	104 (81.3%)	45 (77.6%)	
Squamous cell carcinoma	17 (13.3%)	8 (13.8%)	
Others	7 (5.5%)	5 (8.6%)	
Pathological stage			0.10
0–IA	86 (67.2%)	46 (79.3%)	
IB	24 (18.8%)	10 (17.2%)	
II–III	18 (14.1%)	2 (3.4%)	

Data are expressed as group mean ± standard error (range) or number (%).

BMI: body mass index; yes/no: former and current smoker/never; RUL: right upper lobectomy; RML: right middle lobectomy; RLL: right lower lobectomy; LUL: left upper lobe lobectomy; LLL: left lower lobectomy; VATS: video-assisted thoracoscopic surgery; RATS: robotic-assisted thoracoscopic surgery

**Table 2:** Clinicopathological characteristics after propensity score matching

Characteristic	VATS group (n = 55)	RATS group (n = 55)	*p*-Value
Age (years)	67.8 ± 1.7 (17–87)	69.1 ± 1.2 (47–87)	0.53
Sex, male/female (%)	24 (43.6%)/31 (56.4%)	25 (45.5%)/30 (54.5%)	0.85
Height (cm)	159.5 ± 1.2 (144–182)	158.8 ± 1.2 (142–177)	0.69
Body weight (kg)	61.0 ± 1.6 (42.6–93.5)	59.7 ± 1.6 (36–92.2)	0.54
BMI (kg/m^2^)	24.0 ± 0.5 (17.7–38.2)	23.6 ± 0.5 (14.9–33.0)	0.63
Smoking history, yes/no (%)	29 (52.7%)/26 (47.3%)	27 (49.1%)/28 (50.9%)	0.70
Performance status, 0/1 (%)	53 (96.4%)/2 (3.6%)	54 (98.2%)/1 (1.8%)	0.56
Past medical history			
Hypertension	11 (8.6%)	20 (34.5%)	0.06
Malignant disease	9 (7.0%)	8 (13.8%)	0.79
Arrhythmia	2 (1.6%)	3 (5.2%)	0.65
Ischemic heart disease	4 (3.1%)	2 (3.4%)	0.40
Diabetes mellitus	1 (0.8%)	2 (3.4%)	0.56
Interstitial lung disease	1 (0.8%)	1 (1.7%)	1.00
Cerebrovascular disease	0 (0%)	2 (3.4%)	
Operative time (min)	156 ± 5.9 (90–304)	172 ± 5.7 (84–280)	0.06
Blood loss (mL)	30 ± 7.7 (1–322)	40 ± 12.1 (1–522)	0.45
Lobectomy procedure types			0.90
RUL	21 (38.2%)	22 (40.0%)	
RML	2 (3.6%)	1 (1.8%)	
RLL	16 (29.1%)	13 (23.6%)	
LUL	11 (20.0%)	12 (21.8%)	
LLL	5 (9.1%)	7 (12.7%)	
Postoperative complications			
Pulmonary complications	14 (25.5%)	8 (13.8%)	0.15
Cardiovascular complications	5 (9.1%)	3 (5.2%)	0.46
Histologic type			0.46
Adenocarcinoma	46 (83.6%)	43 (78.2%)	
Squamous cell carcinoma	4 (7.3%)	8 (14.5%)	
Others	5 (9.1%)	4 (7.3%)	
Pathological stage			0.56
0–IA	47 (85.5%)	43 (78.2%)	
IB	6 (10.9%)	10 (18.2%)	
II–III	2 (3.6%)	2 (3.6%)	

Data are expressed as group mean ± standard error (range) or number (%).

BMI: body mass index; yes/no: former and current smoker/never; RUL: right upper lobectomy; RML: right middle lobectomy; RLL: right lower lobectomy; LUL: left upper lobe lobectomy; LLL: left lower lobectomy; VATS: video-assisted thoracoscopic surgery; RATS: robotic-assisted thoracoscopic surgery

### Preoperative pulmonary function

PFT data are summarized in **Table 3**. No significant differences were found between the VATS and RATS groups for any of the evaluated parameters: %predicted forced vital capacity (%FVC), %predicted forced expiratory volume in one second (%FEV1), %predicted peak expiratory flow (%PEF), or %predicted diffusing capacity of the lung for carbon monoxide (%DLco).

**Table 3:** Preoperative pulmonary function test before and after propensity score matching

	Before PSM		*p*-Value	After PSM		*p*-Value
	VATS group (n = 128)	RATS group (n = 58)		VATS group (n = 55)	RATS group (n = 55)	
%FVC	110.3 ± 1.5	110.1 ± 2.1	0.96	111.3 ± 2.3	109.5 ± 2.2	0.58
%FEV1	100.7 ± 1.6	102.4 ± 2.5	0.62	103.0 ± 2.2	101.4 ± 2.5	0.36
%PEF	85.8 ± 1.5	86.4 ± 1.9	0.83	86.2 ± 2.1	86.5 ± 1.9	0.92
%DLco	113.1 ± 2.4	110.5 ± 3.5	0.55	109.2 ± 3.0	111.5 ± 3.6	0.63

Data are expressed as group mean ± standard error.

%FVC: %predicted forced vital capacity; %FEV1: %predicted forced expiratory volume in one second; %PEF: %predicted peak expiratory flow; %DLco: %predicted diffusing capacity of the lung for carbon monoxide; PSM: propensity score matching; VATS: video-assisted thoracoscopic surgery; RATS: robotic-assisted thoracoscopic surgery

### Postoperative pulmonary function

**Fig. 2:**
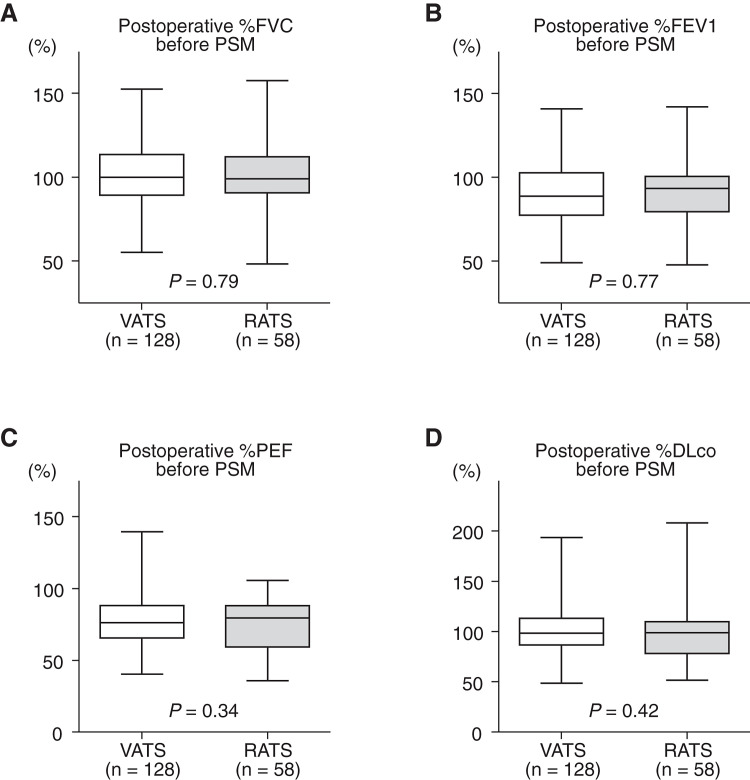
Postoperative pulmonary function test data before PSM. (A) %FVC, (B) %FEV1, (C) %PEF, and (D) %DLco compared between the VATS and RATS groups. No statistically significant differences were observed between groups. PSM: propensity score matching; VATS: video-assisted thoracoscopic surgery; RATS: robotic-assisted thoracoscopic surgery; %FVC: %predicted forced vital capacity; %FEV1: %predicted forced expiratory volume in one second; %PEF: %predicted peak expiratory flow; %DLco: %predicted diffusing capacity of the lung for carbon monoxide

Postoperative PFT data before PSM are presented in **Fig. 2**. There were no significant differences between the VATS and RATS groups in any of the measured parameters:

- %FVC: 100.8% ± 1.6% (VATS) vs. 101.6% ± 2.5% (RATS); *p* = 0.79
- %FEV1: 91.6% ± 1.6% (VATS) vs. 90.7% ± 2.6% (RATS); *p* = 0.77
- %PEF: 78.0% ± 1.7% (VATS) vs. 75.3% ± 2.1% (RATS); *p* = 0.34
- %DLco: 101.3% ± 2.1% (VATS) vs. 98.2% ± 3.4% (RATS); *p* = 0.42

**Fig. 3:**
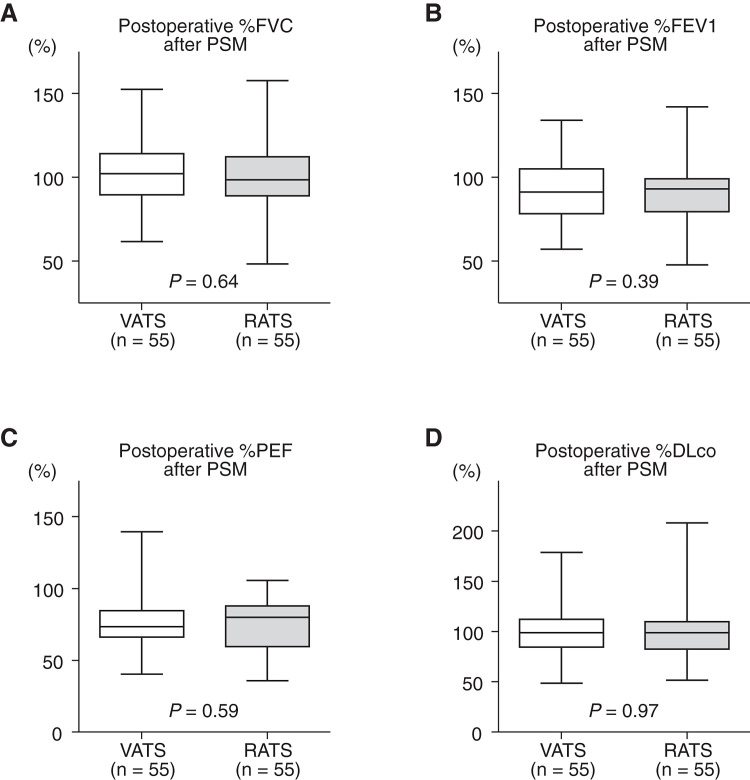
Postoperative pulmonary function test data after PSM. (A) %FVC, (B) %FEV1, (C) %PEF, and (D) %DLco compared between the VATS and RATS groups. No statistically significant differences were observed between the two groups. PSM: propensity score matching; VATS: video-assisted thoracoscopic surgery; RATS: robotic-assisted thoracoscopic surgery; %FVC: %predicted forced vital capacity; %FEV1: %predicted forced expiratory volume in one second; %PEF: %predicted peak expiratory flow; %DLco: %predicted diffusing capacity of the lung for carbon monoxide

Postoperative PFT data after PSM are shown in **Fig. 3**. Again, no statistically significant differences were observed between the groups:

- %FVC: 102.3% ± 2.6% (VATS) vs. 100.5% ± 2.6% (RATS); *p* = 0.64
- %FEV1: 92.8% ± 2.3% (VATS) vs. 89.7% ± 2.6% (RATS); *p* = 0.39
- %PEF: 77.4% ± 2.5% (VATS) vs. 75.6% ± 2.2% (RATS); *p* = 0.59
- %DLco: 98.7% ± 3.0% (VATS) vs. 98.9% ± 3.5% (RATS); *p* = 0.97

## Discussion

In this study, we evaluated 1-year postoperative pulmonary function following RATS and VATS for early-stage NSCLC. After applying PSM to reduce selection bias, no significant differences were observed between the groups in postoperative lung function parameters, including %FVC, %FEV1, %PEF, and %DLco. These findings indicate that both surgical approaches have a comparable impact on long-term pulmonary function.

The present results are consistent with previous reports demonstrating similar perioperative outcomes between RATS and VATS.^1–3)^ Although RATS typically involves five ports, including a 4–5 cm utility incision, and VATS generally uses three ports, both techniques resulted in comparable preservation of pulmonary function at one year. This suggests that the increased number of ports in RATS does not adversely affect long-term pulmonary function. In our RATS technique, all ports other than the utility incision are placed, as much as possible, within the same ICS. In addition, the robotic instruments move around a fixed fulcrum at the chest wall, which may help minimize trauma to the intercostal nerves and muscles. These technical characteristics could partly explain why postoperative pulmonary function was preserved at a level comparable to that after VATS.

Understanding the impact of surgical approaches on postoperative outcomes is particularly important when implementing newer techniques such as RATS. While one prior study reported superior preservation of %FVC and %FEV1 in the RATS group at three months postoperatively,^7)^ the present study extended this observation to one year, based on the notion that pulmonary function generally stabilizes within 6 to 12 months following lung resection.^12)^ When the extent of resection is equivalent, the surgical approach itself may have only a minimal effect on postoperative pulmonary function. However, to the best of our knowledge, this is the first study to comprehensively compare the effects of RATS and VATS on pulmonary function at the one-year time point.

Despite comparable functional outcomes, cost remains a significant consideration when selecting a surgical approach. RATS has been associated with higher costs compared to VATS, which may influence decision-making, particularly in healthcare systems with limited resources. Pan et al. reported that RATS incurred greater lobectomy costs despite achieving improved perioperative outcomes.^13)^ Similarly, Alwatari et al. found that RATS was more costly while yielding complication rates and lengths of stay similar to those of VATS.^3)^ Given the absence of significant differences in pulmonary function outcomes, VATS may represent a more cost-effective approach for lobectomy in patients with early-stage lung cancer. The primary purpose of this study was to demonstrate the functional non-inferiority of RATS compared with VATS in standard lobectomy procedures. Confirming that RATS achieves functional outcomes equivalent to those of VATS in standard lobectomy procedures is an important prerequisite for evaluating the potential advantages of RATS in more complex or technically demanding cases. Furthermore, RATS may retain value as a technological platform, offering advantages in complex procedures and facilitating future developments in thoracic surgery.

This study has several limitations. First, the retrospective design introduces inherent potential for bias. Second, the relatively small sample size after PSM may have limited the ability to detect subtle differences. Furthermore, functional exercise assessments, such as the 6-minute walk test, and quality-of-life evaluations were not included, which may have provided a more comprehensive view of postoperative recovery.

## Conclusion

RATS and VATS yield comparable one-year postoperative pulmonary function following lobectomy for early-stage NSCLC. Although RATS may offer technical advantages, its higher cost may not be justified in the absence of superior functional outcomes. Future studies incorporating functional and quality-of-life measures are warranted to further elucidate the relative benefits of each surgical approach.

## Declarations

### Data availability

The data that support the findings of this study are available from the corresponding author upon reasonable request.
